# Biochar and its combination with nitrogen fertilisation altered soil organic matter, humic substances, and soil structure: short-term versus long-term changes

**DOI:** 10.1007/s10653-025-02853-7

**Published:** 2025-10-28

**Authors:** Vladimír Šimanský, Elżbieta Wójcik-Gront, Sanandam Bordoloi, Ján Horák

**Affiliations:** 1 Institute of Agrochemistry and Soil Science, Faculty of Agrobiology and Food Resources, Slovak University of Agriculture, Nitra, 949 76 Slovakia; 2 Department of Biometry, Institute of Agriculture, Warsaw University of Life Sciences—SGGW, Nowoursynowska Str. 159, 02-776 Warsaw, Poland; 3 Department of Civil Engineering, School of Engineering, Aalto University, Espoo, Finland; 4 Institute of Landscape Engineering, Faculty of Horticulture and Landscape Engineering, Slovak University of Agriculture, Nitra, 949 76 Slovakia

**Keywords:** Biochar, Fulvic acids, Humic substances, Soil organic matter, Soil aggregates

## Abstract

Biochar (B), particularly when combined with nitrogen (N) fertilisation, can significantly influence soil quality and fertility. However, its long-term effects on soil organic matter (SOM), humic substances (HS), and soil structure remain insufficiently understood. This study examined the impacts of biochar applied at 0, 10, and 20 t ha⁻^1^ (B0, B10, B20), in combination with two levels of N fertilization (N1, N2), on a silty loam Haplic Luvisol over short-term (1 year) and long-term (9 years) periods at the Slovak University of Agriculture experimental site (Nitra, Slovakia). After 1 year, biochar treatments increased soil organic carbon (Corg) by up to 51% (B20N1) compared with the control (B0N0), but significantly reduced the extraction of humic substances, particularly in B20N1. After 9 years, Corg contents were relatively balanced across treatments, but B20N2 exhibited a marked increase in HS content and a 33% reduction in microaggregates relative to B0N0, indicating the formation of larger macroaggregates. Principal component and correlation analyses revealed time-dependent changes in the relationships between SOM, HS, and aggregate size fractions. Importantly, almost no consistent correlation was observed between Corg and aggregate size fractions, suggesting that the quality and chemical characteristics of humic substances, especially their aromatic and condensed nature, play a more critical role in soil structure formation than total carbon content alone. This study provides new evidence that biochar’s long-term impact on soil structure is mediated not by immediate increases in carbon levels, but by gradual improvements in humus chemistry and aggregate dynamics. Our findings challenge conventional assessments of soil amendments based solely on carbon metrics and highlight the need to consider humic substance quality as a key driver of soil structural resilience.

## Introduction

Soil structure and humic substances are fundamental components of agricultural soils, and their formation, dynamics, and interactions have long been the focus of soil science. Different perspectives and approaches have been used to describe these properties. For example, Amezketa (1999) and Bronick and Lal (2005), in their reviews on soil structure, considered a broad range of environmental and anthropogenic factors and their interactions, emphasising the role of soil aggregates and their stability. Many recent studies continue in this direction, exploring aggregate stability and its measurement (Guo et al., 2024; Mamedov et al., 2021; Siebers et al., 2024; Šimanský, 2014; Šimanský et al., 2023a, 2023b). In contrast, Letey (1991) proposed a different perspective by focusing on the arrangement and evolution of soil pores, highlighting their unique structural architecture over time. Regardless of the approach, soil structure is widely recognised as a fundamental physical property that warrants close attention (Blume et al., 2016; Foth, 1990; Lal & Shukla, 2004).

Similarly, the study of humus, despite more than two centuries of scientific investigation, continues to evolve, reflecting shifts in theory and methodology (Lehmann & Kleber, 2015; Stevenson, 1994). Humus is a vital component of soil organic matter (SOM) (Stevenson, 1994; Weil & Brady, 2017), playing key roles in soil formation and influencing physical, chemical, nutritional, and biological properties (Mu et al., 2024; Poláková et al., 2018). The content and quality of humic substances (HS) are strongly linked to the development and stability of soil structure (Poláková et al., 2018). However, knowledge gaps remain concerning how soil structure and humic substances respond to biochar application, biochar-derived substrates, or their combinations with mineral fertilisers (Juriga et al., 2019; Mierzwa-Hersztek et al., 2018; Zhang et al., 2014).

Biochar is a porous, highly aromatic, and largely insoluble material produced by the pyrolysis of lignocellulosic biomass and plant residues under low-oxygen or anoxic conditions (Hansen et al., 2016). Rich in stable carbon, biochar resists decomposition and persists in soils over long timescales, contributing to carbon sequestration (Hossain et al., 2020; Šrank & Šimanský, 2020). Its high specific surface area, microporous structure (Chintala et al., 2014), low bulk density (Głąb et al., 2016), and abundance of functional groups (e.g. phenolic, alcoholic hydroxyl, and carbonyl groups) (Głąb et al., 2016; Xu et al., 2014) enhance a range of soil properties. These include the immobilisation of harmful substances such as heavy metals (Shen, 2024), regulation of water dynamics (Igaz et al., 2018; Sharma, 2024), nutrient availability (Chen et al., 2018; Li et al., 2020), cation exchange capacity and sorption properties (Hossain et al., 2020; Igaz et al., 2018), and the reduction of bulk density (Głąb et al., 2016). Biochar application can also influence soil physical properties, improving aggregation and creating a more stable and porous soil matrix (Juriga & Šimanský, 2018; Zhang et al., 2022). This improved structure reduces compaction, particularly in clay-rich soils (Blanco-Canqui, 2021), and promotes root growth and microbial activity, thereby supporting higher crop yields.

While biochar’s potential to improve soil carbon content and structure is well established, few studies have investigated its *long-term* interactions with humic substances and soil aggregation dynamics under field conditions. This study addresses this gap by comparing short-term (1 year) and long-term (9 years) responses in a temperate agricultural soil.

In this study, we tested three hypotheses. First (H1), given that humus formation is a slow, long-term process, we hypothesised that the effects of biochar and its combination with N fertilisation on humus development would become more pronounced over time. Second (H2), we expected that prolonged biochar presence would improve soil structure by reducing microaggregates and increasing favourable macroaggregates in the 0.5–3 mm size range. Third (H3), we hypothesised that changes in SOM and HS would directly affect the size distribution of soil aggregates. Based on these hypotheses, the objectives of this study were:

(i) To quantify the effects of biochar and its combination with nitrogen fertilisation on the content and quality of humic substances and soil structure, and.
(ii) To assess how the relationships between humic substances and soil structure evolve over time.

## Material and methods

### ***Site ***description

The long-term field experiment was conducted at the experimental site of the Slovak University of Agriculture in Nitra (Dolná Malanta; 48° 10′ 00″ N, 18° 09′ 00″ E), located in the north-eastern Danubian Lowland and the western Žitava Upland, near the lower basin of the Selenec stream, approximately 4 km east of Nitra, Slovakia. The site lies in a warm maize-growing region on flat terrain with a gentle south-western slope at an elevation of 170–180 m above sea level. Geologically, it is situated at the boundary between the crystalline–Mesozoic massif of the Tribeč Mountains and the Žitava Upland. The parent material consists mainly of eluvial–deluvial sediments from the Tribeč Mountains, mixed in places with loess sediments from the Žitava Upland.

The soil is classified as a Haplic Luvisol with a silt loam texture in the A-horizon. Its particle size composition comprises 360.4 g kg⁻^1^ sand, 488.3 g kg⁻^1^ silt, and 151.3 g kg⁻^1^ clay. The soil has a slightly acidic reaction (pH 5.71), low soil organic carbon content (9.13 g kg⁻^1^), a moderately low cation exchange capacity (142 mmol p⁺ kg⁻^1^), and a saturated sorption complex of 85%. The site is located in a very warm and dry agro-climatic region, with a mean annual precipitation of 559 mm and a mean annual temperature of 10.7 °C (1991–2020 climatological norm).

### Experimental design

The biochar field trial was established in spring 2014 before sowing spring barley and has been ongoing since then. The crop rotation during the experimental period was as follows: spring barley (2014), maize (2015), spring wheat (2016), maize (2017), spring barley (2018), maize (2019), peas (2020), winter wheat (2021), maize (2022), and spring barley (2023).

The experiment included nine treatments (Table 1) arranged in a randomised block design with three replicates per treatment. Each plot measured 6 × 4 m and was separated by a 1 m buffer strip. Biochar was applied to the soil surface in spring 2014 at rates of 10 and 20 t ha⁻^1^ (B10 and B20), manually distributed and incorporated into the soil to a depth of 10 cm using a combinator. Nitrogen fertilisation was applied at two levels: N1 as the standard rate calculated according to crop requirements and Slovak agronomic recommendations, and N2 as a treatment 100% higher than N1.

**Table 1:** Experimental design for the current study

Sl. No	Designation	Description
1	B0N0	No biochar, no nitrogen
2	B10N0	Biochar at a rate of 10 t ha^−1^ and no nitrogen
3	B20N0	Biochar at a rate of 20 t ha^−1^ and no nitrogen
4	B10N1	Biochar at a rate of 10 t ha^−1^ and a first level of N fertilization
5	B20N1	Biochar at a rate of 20 t ha^−1^ and a first level of N fertilization
6	B10N2	Biochar at a rate of 10 t ha^−1^ and a second level of N fertilization
7	B20N2	Biochar at a rate of 20 t ha^−1^ and a second level of N fertilization

Nitrogen was applied during the growing season in two or three split applications, depending on the total rate. Over the course of the experiment, nitrogen application ranged from 30 to 160 kg N ha⁻^1^ for N1 and from 45 to 240 kg N ha⁻^1^ for N2. For example, in 2015 the doses were 160 and 240 kg N ha⁻^1^ for N1 and N2, respectively (applied on 24 April and 5 August as LAV 27), while in 2023 the rates were 40 kg N ha⁻^1^ (N1) and 80 kg N ha⁻^1^ (N2) (applied on 4 March as LAD 27, 28 April as LAD 27, and 12 May as DASA 26). No other fertilisers were applied in the selected treatments. Throughout the experiment, conventional agronomic practices were followed, including weed, pest, and disease control. The soil was not ploughed; instead, shallow tillage (discing or surface cultivation to a maximum depth of 15 cm) was used.

### Biochar and nitrogen mineral fertilizers

The biochar used in this study was produced from cereal husks and paper industry sludge by pyrolysis at 550 °C for 30 min. It contained 53.1% total carbon, 1.4% total nitrogen, 57 g kg⁻^1^ calcium, 3.9 g kg⁻^1^ magnesium, 15 g kg⁻^1^ potassium, 0.7 g kg⁻^1^ sodium, and 38.3% ash. The biochar had a specific surface area of 21.7 m^2^ g⁻^1^, a pH of 8.8, and a particle size range of 1–5 mm. Nitrogen was supplied using the following commercial fertilisers: LAV 27 (ammonium calcium nitrate, 27% total N: 13.5% nitrate N, 13.5% ammonium N, plus 20% CaCO₃), LAD 27 (ammonium nitrate with dolomite, 27% total N, plus 4.1% total MgO and 1% water-soluble MgO), and DASA 26 (ammonium nitrate with sulphur, 26% total N: 18.5% ammonium N, 7.5% nitrate N, plus 32.5% water-soluble sulphur oxide).

### Soil sampling and analysis

Soil samples were collected from the 0 to 20 cm layer at monthly intervals from April to September in 2015 and from April to July in 2023. Samples were taken from each replicate of every treatment using a spade to minimise aggregate disturbance, and roots and coarse organic debris were removed. Dried samples were sieved to determine the size distribution of soil aggregates. For each sample, 200 g of air-dried soil was placed on a stack of sieves with mesh sizes of 3, 0.5, and 0.25 mm and shaken for 10 min using an AS 200 sieve shaker. The weight of aggregates retained on each sieve was measured and expressed as a percentage of total soil mass. Four dry soil aggregate (DSA) size fractions were distinguished: > 3 mm, 3–0.5 mm, 0.5–0.25 mm, and < 0.25 mm. Aggregates > 0.25 mm were classified as macroaggregates, while those < 0.25 mm were considered microaggregates. Macroaggregates of 0.5–3 mm are regarded as agronomically valuable (Fulajtár, 2006; Šimanský et al., 2023a).

Samples for SOM and humus analyses were homogenised, ground, and sieved (< 0.25 mm). Soil organic carbon (Corg) was determined by wet oxidation with 0.07 mol L⁻^1^ H₂SO₄ and K₂Cr₂O₇, followed by titration with Mohr’s salt (Dzadowiec & Gonet, 1999). Humic substances (HS), humic acids (HA), and fulvic acids (FA) were extracted with 0.01 mol L⁻^1^ Na₄P₂O₇·10 H₂O and 0.1 mol L⁻^1^ NaOH (Belchikova and Kononova method; Dzadowiec & Gonet, 1999). The colour quotients of HS (Q^₄/₆^_HS_) and HA (Q^₄/₆^_HA_) were measured at 465 and 650 nm using a Jenway 6400 spectrophotometer. The degree of humification (DH) of SOM was calculated as:

1 $$DH=\frac{{C}_{HA}}{Corg}\times 100$$

where *C*_*HA*_ denotes the carbon of humic acids, *Corg* is the content of soil organic carbon.

### Statistical analysis

A two-way ANOVA was initially applied to test the effects of year and treatment on the measured variables. Subsequently, for each year, one-way ANOVA was used with treatment as the main factor, treating sampling time (April–September for 2015 and April–July for 2023) as replicates. Analysing each year separately avoids assuming identical treatment effects across years and enables a more nuanced interpretation of temporal dynamics. Pearson correlation analysis was used to examine relationships between SOM, humic substances, and aggregate size fractions within each treatment for both years. Principal component analysis (PCA) was performed to reduce data dimensionality while retaining the most important variance. The first two principal components (PC1 and PC2) were selected for interpretation and visualised in a biplot, illustrating the relationships between treatments and variables and revealing major patterns in the dataset.

## Results

### Soil organic matter and humic substances

**Fig. 1:**
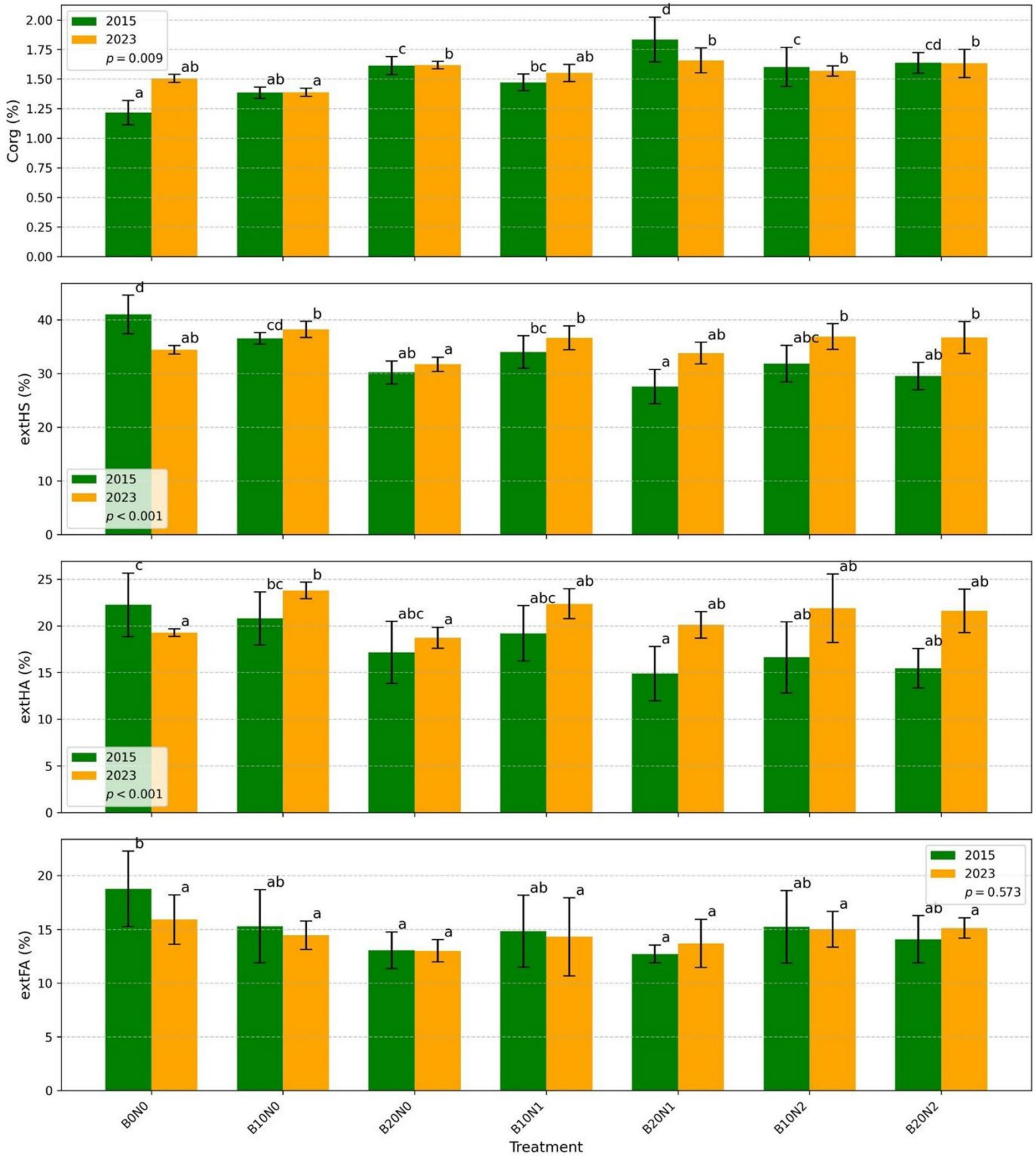
Comparison of soil organic carbon (Corg) and humic substance fractions between 2015 (green bars) and 2023 (orange bars) across different treatments. The bar plots display the mean values (± standard deviation) for soil organic carbon (Corg, %), extractable humic substances (extHS, %), extractable humic acids (extHA, %), and extractable fulvic acids (extFA, %). Treatments include B0N0, B10N0, B20N0, B10N1, B20N1, B10N2, and B20N2. Different letters above bars indicate statistically significant differences between treatments within each year based on Tukey’s HSD test (*p* < 0.05)

Across all treatments, soil organic carbon (Corg) contents were generally higher (except B20N1, B10N2) in 2023 than in 2015 (Fig. 1). In the control treatment (B0N0), Corg increased from 1.22% in 2015 to 1.51% in 2023. In 2015, biochar application and its combination with nitrogen (N) fertilisation increased Corg content by 32% in B20N0, 21% in B10N1, 51% in B20N1, 31% in B10N2, and 34% in B20N2 compared with B0N0. The highest increase occurred in B20N1. By 2023, differences in Corg among treatments were smaller and relatively balanced.

The extraction of humic substances (extHS) decreased in 2015 compared with the control, with the lowest values observed in B20N1. In 2023, extHS values were higher than in 2015 but showed no significant differences between treatments. Humic acid extraction (extHA) followed a similar pattern. In 2015, significant decreases were observed in B20N1, B10N2, and B20N2. By 2023, extHA increased significantly only in B10N0, while other treatments remained similar to the control. Fulvic acid extraction (extFA) decreased in 2015, with a significant reduction in B20N0 and in B20N1, but showed no significant differences between treatments in 2023. The degree of humification (DH) ranged from 15 to 24% in 2015 and from 20 to 24% in 2023. Primary organic matter predominated over humic substances in all treatments.

**Fig. 2:**
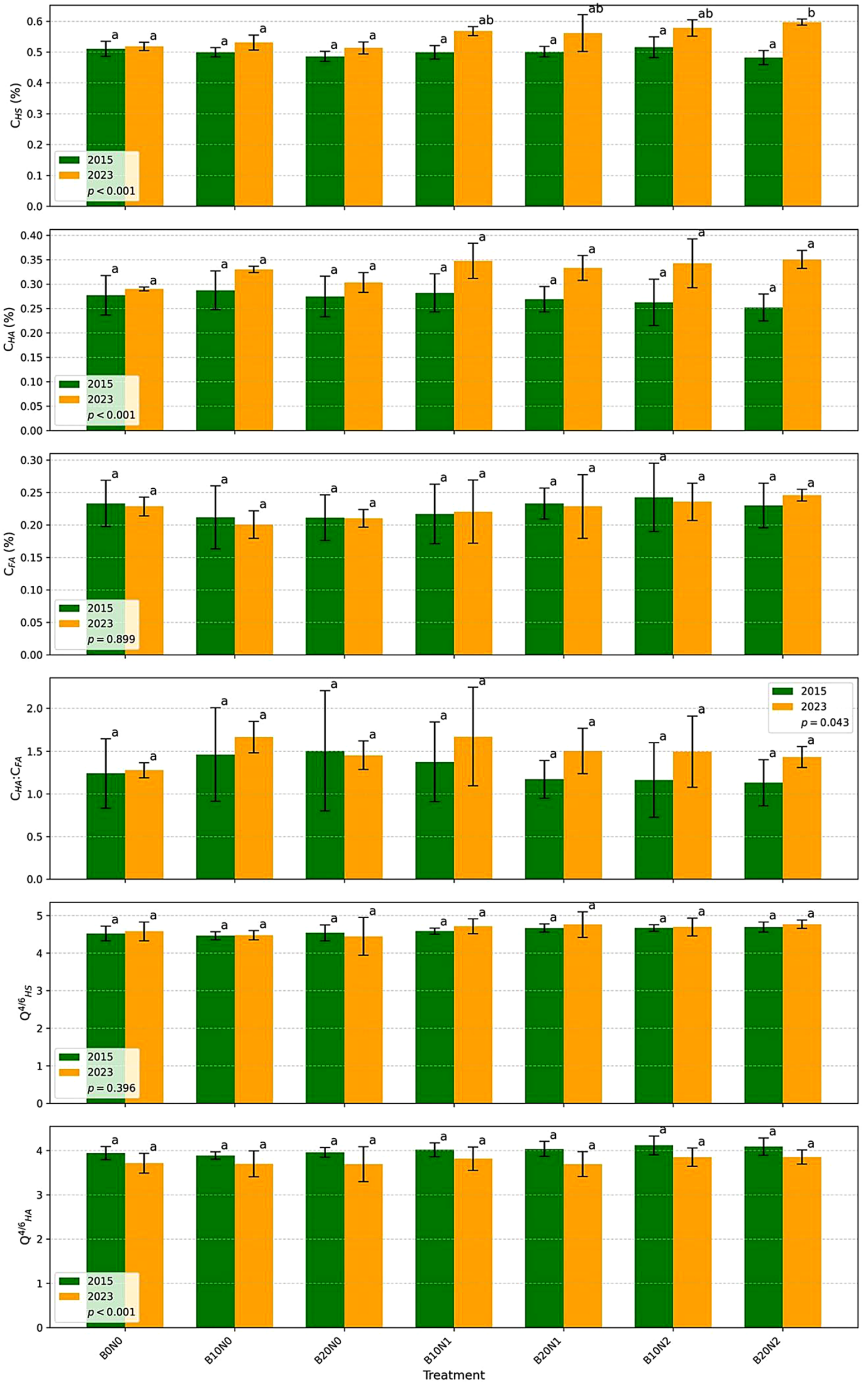
Comparison of humus-related parameters between 2015 (green bars) and 2023 (orange bars) across different treatments. The bar plots display the mean values (± standard deviation) for carbon in humic substances (C_HS_, %), carbon in humic acids (C_HA_, %), carbon in fulvic acids (C_FA_, %), C_HA_:C_FA_ ratio, color quotient of humic substances (Q^4/6^_HS_), and color quotient of humic acids (Q^4/6^_HA_). Treatments include B0N0, B10N0, B20N0, B10N1, B20N1, B10N2, and B20N2. Different letters above bars indicate statistically significant differences between treatments within each year based on Tukey’s HSD test (*p* < 0.05)

Humic substances (C_HS_) did differ significantly between 2015 and 2023. A moderate increase of C_HS_ was in B20N2 in 2023. The C_HA_:C_FA_ ratio remained above 1 across all treatments and years, with no significant changes. Colour quotient values of humic substances and humic acids indicated highly humified and mature organic matter with a high degree of condensation and aromaticity. The molecular weight and degree of condensation of humic substances remained unchanged (Fig. 2).

### Soil structure

**Fig. 3:**
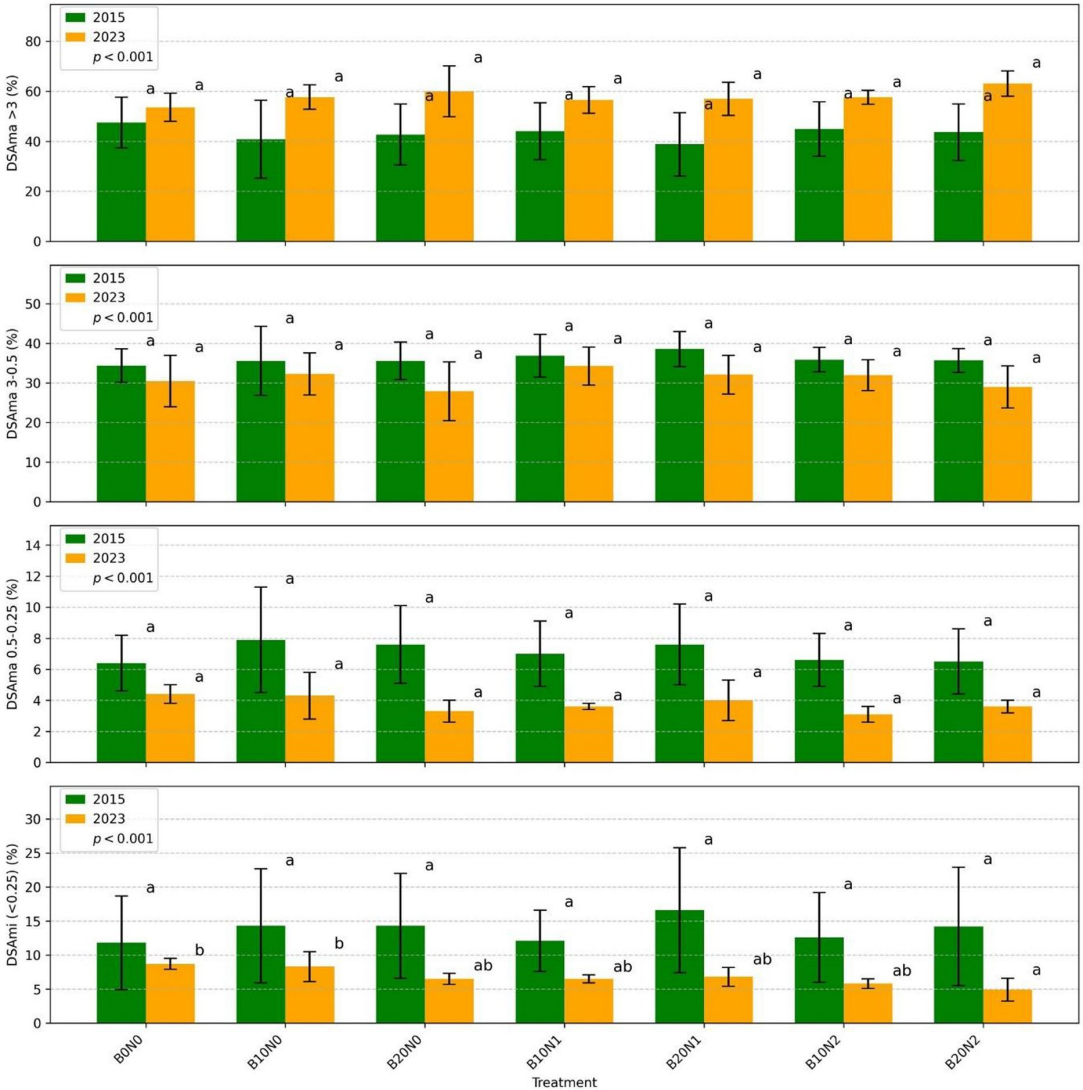
Bar plots, each representing different variables (DSAma and DSAmi) across multiple treatments (B0N0, B10N0, B20N0, B10N1, B20N1, B10N2, B20N2) for the years 2015 (green bars) and 2023 (yellow bars). The error bars indicate standard deviations. Letters above the bars denote homogeneous groups for the treatments in a specific year. Treatments sharing the same letter are not significantly different from each other within that year, according to the Tukey procedure at a significance level of 0.05

No statistically significant effects of biochar or nitrogen fertilisation on dry soil macroaggregates (DSAma) were observed in either year. DSAma > 3 mm increased significantly in 2023 compared with 2015 (*p* < 0.001), whereas DSAma 0.25–3 mm were significantly higher in 2015 than in 2023 (*p* < 0.001). Microaggregate (DSAmi) contents were significantly lower in 2023 compared with 2015 (*p* < 0.001). In 2015, biochar and its combinations with nitrogen had no effect on DSAmi. In 2023, microaggregate contents decreased by 33% in B20N2 compared with B0N0 and by 30% compared with B10N0. A decreasing trend was also observed in B20N0, B10N1, B20N1, and B10N2. This reduction did not correspond to a significant increase in macroaggregates in the 0.5–3 mm range (Fig. 3).

### Relationships between SOM, humic substances, and soil structure

**Fig. 4:**
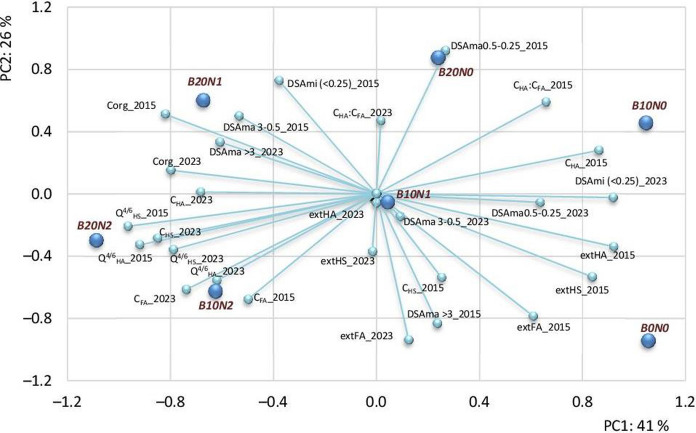
Principal Component Analysis (PCA) biplot illustrating the relationships between soil properties and experimental treatments in 2015 and 2023. Vectors represent soil organic matter (SOM) parameters, humic substances, and soil structural properties. The positions of treatments (B0N0, B10N0, B20N0, B10N1, B20N1, B10N2, B20N2) indicate how they associate with different variables across years

No statistically significant correlations were found between Corg and individual aggregate size fractions (DSAma or DSAmi) in either 2015 or 2023 with a few exceptions (in 2023). Treatments differed in the relationships among SOM, humic substances, and aggregate size fractions, and these relationships changed between 2015 and 2023 (Table 2). B10N0 was associated with higher values of DSAma (0.5–0.25 mm) and C_HA_ in 2015, while B20N2 showed opposite patterns (Fig. 4). The total number of significant correlations was higher in 2015 than in 2023 and varied substantially among treatments.

**Table 2:**
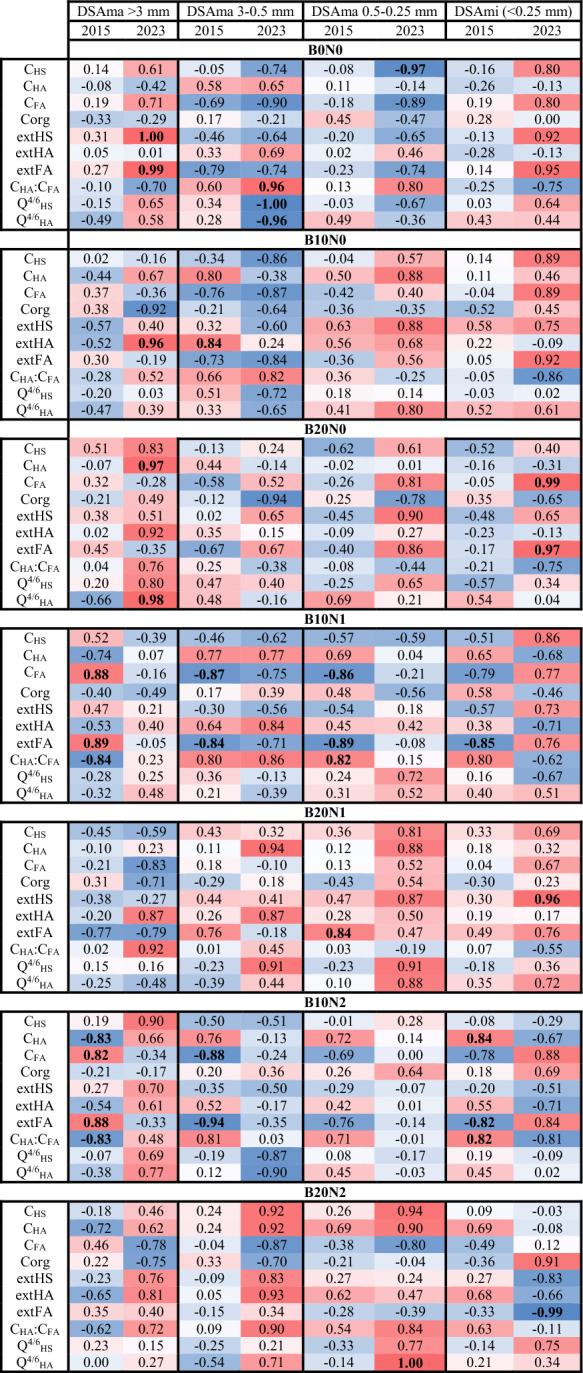
Results of correlation analysis according to Pearson procedure

The red color of cells means positive correlations, and the blue one indicates negative correlations. Bold font was used to indicate significant correlations at *p* < 0.05

## Discussion

The results of this study demonstrate that biochar and its combination with nitrogen fertilisation influence soil organic matter (SOM), humic substances, and soil structure in a time-dependent manner. The initial increase in soil organic carbon (Corg) following biochar application is consistent with previous findings that biochar contains a large fraction of stable carbon that resists decomposition and persists in soils over the long term (Hossain et al., 2020; Šrank & Šimanský, 2020). The substantial increase in Corg observed in B20N1 in 2015 suggests a strong negative priming effect, whereby biochar reduces the mineralisation of native SOM and enhances carbon sequestration (Kalu et al., 2024). The adsorption of labile organic compounds onto biochar surfaces (Jones et al., 2012) may further contribute to higher Corg levels. By 2023, differences in Corg among treatments had diminished, suggesting that the influence of biochar on total soil carbon decreased over time as biochar particles became incorporated into soil aggregates and earthworm casts (Šimanský, 2016; Šimanský et al., 2019). This shift reflects the long-term stabilisation of biochar carbon and the increasing importance of broader soil management practices in determining SOM content. The decline in humic substance extraction (extHS, extHA, extFA) in 2015 despite increased Corg indicates that biochar initially reduces SOM mineralisation and humification. Biochar’s low chemical reactivity and stable structure (Gupta & Germida, 2015; Nguyen et al., 2010) slow down the transformation of organic matter, resulting in reduced formation of humic substances. Similar findings have been reported by Mierzwa-Hersztek et al. (2018), who observed that biochar can initially decrease humic substance formation even as total carbon increases. Over time, however, the effects of biochar became less pronounced, and by 2023 humic substance levels were more stable and balanced across treatments. This supports the view that humus formation is a slow, long-term process (Stevenson, 1994; Weil & Brady, 2017), and biochar’s influence on humification becomes evident only after several years.

The consistently high C_HA_:C_FA_ ratio (> 1) across treatments indicates that humus quality remained favourable throughout the experiment. A slight shift towards higher C_HA_ proportions suggests a gradual increase in humic substance aromaticity and condensation, which enhances soil structure stability (Weber, 2020). Conversely, increased nitrogen availability tended to reduce the C_HA_:C_FA_ ratio, likely due to enhanced decomposition of labile organic matter and increased fulvic acid formation. The type and composition of biochar strongly influence these processes (Zhao et al., 2017).

The structural effects of biochar were subtle and emerged only after several years. The lack of significant differences in macroaggregates after 1 year is consistent with previous findings that biochar-induced structural improvements often manifest over longer timescales (Jien & Wang, 2013). Several mechanisms contribute to these effects. Biochar’s reactive surface functional groups form organo-mineral complexes with clay, forming the foundation of microaggregates (Brodowski et al., 2006). Fungal hyphae colonising biochar pores can further stabilise aggregates by binding soil particles (Cross et al., 2014). Combined application with nitrogen can accelerate SOM mineralisation and stimulate microbial activity, leading to the production of extracellular polymeric substances that promote aggregate formation (Ali et al., 2020; Costa et al., 2018). Climatic factors also played a significant role in aggregate dynamics. Wetting–drying and freeze–thaw cycles strongly influence soil structure (Blume et al., 2016; Foth, 1990; Lal & Shukla, 2004), and differences in precipitation and temperature between 2015 and 2023 likely contributed to the observed changes. The reduction in microaggregate content and the corresponding increase in larger aggregates in 2023, particularly in B20N2, suggest that biochar facilitated the bonding of microaggregates into larger structural units. However, this did not lead to a significant increase in the agronomically most valuable macroaggregate size fraction (0.5–3 mm), indicating that additional factors such as biological activity and climate remain critical. The absence of significant correlations between Corg and aggregate size fractions indicates that total carbon content alone does not determine soil structure. Instead, the quality and composition of SOM, especially the chemical characteristics of humic substances, appear more influential (Oades, 1984). Some organic compounds, such as fulvates, citrates, and oxalates, disperse clay particles, whereas aromatic acids promote flocculation and aggregation (Itami & Kyuma, 1995). This is consistent with the present findings, where lower extFA and C_FA_ contents in treatments with 10 t ha⁻^1^ biochar and nitrogen were associated with increased aggregate formation.

The relationships between SOM, humic substances, and soil structure varied across treatments and over time, reflecting complex interactions between biological, chemical, and physical processes. The greater number of significant correlations in 2015 suggests that microbial and chemical processes played a larger role shortly after biochar application, while physical and climatic factors became more dominant over time.

## Conclusions

This long-term field experiment shows that the effects of biochar and nitrogen fertilisation on soil organic matter, humic substances, and soil structure are strongly time-dependent. Biochar increased soil organic carbon shortly after application, but treatment differences diminished over 9 years, indicating that its direct effect on carbon content is short-lived. More importantly, biochar influenced the quality of humic substances rather than their quantity. Although humic substance extraction initially declined, humus quality improved over time, with a consistently high C_HA_:C_FA_ ratio and increased aromaticity of humic acids.

Changes in soil structure were subtle and appeared only after several years. A significant reduction in microaggregates, especially in treatments with high biochar rates and nitrogen, suggests gradual formation of larger aggregates, although agronomically valuable macroaggregates did not increase substantially. The key finding is that biochar’s long-term benefits arise not from carbon accumulation but from qualitative changes in humic substances that enhance soil structural stability. Future research should focus on the mechanisms linking humus chemistry, microbial processes, and aggregation to optimise biochar use for sustainable soil management.

## Data Availability

No datasets were generated or analysed during the current study.

## References

[CR1] Ali, I., Ullah, S., He, L., Zhao, Q., Iqbal, A., Wei, S., Shah, T., Ali, N., Bo, Y., Adnan, M., & Amanullah Jiang, L. (2020). Combined application of biochar and nitrogen fertilizer improves rice yield, microbial activity and N-metabolism in a pot experiment. *PeerJ,**8*, Article e10311. 33240639 10.7717/peerj.10311 PMC7668215

[CR2] Amezketa, E. (1999). Soil aggregate stability: A review. *Journal of Sustainable Agriculture,**14*, 83–151.

[CR3] Blanco-Canqui, H. (2021). Does biochar application alleviate soil compaction? Review and data synthesis. *Geoderma,**404*, Article 115317.

[CR4] Blume, H. P., Brümmer, G. W., Fleige, H., Horn, R., Kandeler, E., Kögel-Knabner, I., Kretzschmar, R., Stahr, K., & Wilke, B. M. (2016). *Soil science*. Springer.

[CR5] Brodowski, S., John, B., Flessa, H., & Amelung, W. (2006). Aggregate-occluded black carbon in soil. *European Journal of Soil Science,**57*, 539–546.

[CR6] Bronick, C. J., & Lal, R. (2005). Soil structure and management: A review. *Geoderma,**124*, 3–22.

[CR7] Chen, M., Alim, N., Zhang, Y., Xu, N., & Cao, X. (2018). Contrasting effects of biochar nanoparticles on the retention and transport of phosphorus in acidic and alkaline soils. *Environmental Pollution,**239*, 562–570. 29698906 10.1016/j.envpol.2018.04.050

[CR8] Chintala, R., Owen, R. K., Kumar, S., Schumacher, T. E., & Malo, D. (2014). Biochar impacts on denitrification under different soil water contents. *World Congress of Soil Science,**6*, 157.

[CR9] Costa, O. Y. A., Raaijmakers, J. M., & Kuramae, E. E. (2018). Microbial extracellular polymeric substances: Ecological function and impact on soil aggregation. *Frontiers in Microbiology,**9*, 1636. 30083145 10.3389/fmicb.2018.01636 PMC6064872

[CR10] Cross, A., Zwart, K., Shackley, S., & Ruysschaert, G. (2014). The role of biochar in agricultural soils. In S. Shackley, G. Ruysschaert, K. Zwart, & B. Glaser (Eds.), *Biochar in European soils and agriculture, science and practice* (pp. 73–98). Routledge.

[CR11] Dziadowiec, H., & Gonet, S. S. (1999). *Methodical guidebook for soil organic matter studies*. Polish Society of Soil Science.

[CR12] Foth, H. D. (1990). *Fundamentals of soil science*. Wiley.

[CR13] Fulajtár E (2006) *Physical properties of soil*. VÚPOP, Bratislava, Slovakia. (in Slovak).

[CR14] Głąb, T., Palmowska, J., Zaleski, T., & Gondek, K. (2016). Effect of biochar application on soil hydrological properties and physical quality of sandy soil. *Geoderma,**281*, 11–20.

[CR15] Guo, W., Zhou, Y. P., Xu, J. S., Li, D. D., Chen, M. Q., Wang, Q. X., Zhou, T. T., Zhang, J. B., & Zhao, B. Z. (2024). Straw management and fertilization improve soil aggregate stability by inducing biological binding agents and specific keystone genera. *Pedosphere*. 10.1016/j.pedsph.2024.10.005

[CR16] Gupta, V. V., & Germida, J. J. (2015). Soil aggregation: Influence on microbial biomass and implications for biological processes. *Soil Biology & Biochemistry,**80*, A3–A9.

[CR17] Hansen, V., Müller-Stöver, D., Munkholm, L. J., Peltre, C., Hauggaard-Nielsen, H., & Jensen, L. S. (2016). The effect of straw and wood gasification biochar on carbon sequestration, selected soil fertility indicators and functional groups in soil: An incubation study. *Geoderma,**269*, 99–107.

[CR18] Hossain, M. Z., Bahar, M. M., Sarkar, B., Donne, S. W., Ok, Y. S., Palansooriya, K. N., Kirkham, S., Chowdhury, M. B., & Bolan, N. (2020). Biochar and its importance on nutrient dynamics in soil and plant. *Biochar*. 10.1007/s42773-020-00065-z

[CR19] Igaz, D., Šimanský, V., Horák, J., Kondrlová, E., Domanová, J., Rodný, M., & Buchkina, N. (2018). Can a single dose of biochar affect selected soil physical and chemical characteristics? *Journal of Hydrology and Hydromechanics,**66*, 421–428.

[CR20] Itami, K., & Kyuma, K. (1995). Dispersion behaviour of soils from reclaimed lands with poor soil physical properties and their characteristics with special reference to clay dispersion. *Soil Science and Plant Nutrition,**41*, 45–54.

[CR21] Jien, S. H., & Wang, Ch. S. (2013). Effects of biochar on soil properties and erosion potential in a highly weathered soil. *CATENA,**110*, 225–233.

[CR22] Jones, D. L., Rousk, J., Edwards-Jones, G., De Luca, T. H., & Murphy, D. V. (2012). Biochar mediated changes in soil quality and plant growth in a three-year field trial. *Soil Biology & Biochemistry,**45*, 113–124.

[CR23] Juriga, M., & Šimanský, V. (2018). Effect of biochar on soil structure—Review. *Acta Fytotechnica Et Zootechnica,**21*, 11–19.

[CR24] Juriga, M., Šimanský, V., Horák, J., Kondrlová, E., Igaz, D., Polláková, N., Buchkina, N., & Balashov, E. (2019). The effect of different rates of biochar and biochar in combination with N fertilizer on the parameters of soil organic matter and soil structure. *Journal of Ecological Engineering,**19*, 153–161.

[CR25] Kalu, S., Seppänen, A., Mganga, K. Z., Sietiö, O. M., & Karhu, K. (2024). Biochar reduced the mineralization of native and added soil organic carbon: Evidence of negative priming and enhanced microbial carbon use efficiency. *Biochar,**6*, 7.

[CR26] Lal, R., & Shukla, M. K. (2004). *Principles of soil physics*. Marcel Dekker.

[CR27] Lehmann, J., & Kleber, M. (2015). The continuous nature of soil organic matter. *Nature,**528*, 60–68. 26595271 10.1038/nature16069

[CR28] Letey, J. (1991). The study of soil structure-Science or art. *Australian Journal of Soil Research,**29*, 699–707.

[CR29] Li, S., Yang, F., Li, J., & Cheng, K. (2020). Porous biochar-nanoscale zero-valent iron composites: Synthesis, characterization and application for lead ion removal. *Science of the Total Environment,**746*, Article 141037. 32745850 10.1016/j.scitotenv.2020.141037

[CR30] Mamedov, A. I., Fujimaki, H., Tsunekawa, A., Tsubo, M., & Levy, G. J. (2021). Structure stability of acidic Luvisols: Effects of tillage type and exogenous additives. *Soil & Tillage Research,**206*, Article 104832.

[CR31] Mierzwa-Hersztek, M., Gondek, K., Kopieć, M., & Ukalska-Jaruga, A. (2018). Biochar changes in soil based on quantitative and qualitative humus compounds parameters. *Soil Sci Ann,**69*, 234–242.

[CR32] Mu, D., Mu, L., Geng, X., Mohamed, T. A., & Wei, Z. (2024). Evolution from basic to advanced structure of fulvic acid and humic acid prepared by food waste. *International Journal of Biological Macromolecules,**256*, Article 128413. 38029895 10.1016/j.ijbiomac.2023.128413

[CR33] Nguyen, B. T., Lehmann, J., Hockaday, W. C., Jo, S. S., & Masiello, C. A. (2010). Temperature sensitivity of black carbon decomposition and oxidation. *Environmental Science & Technology,**44*, 3324–3331. 20384335 10.1021/es903016y

[CR34] Oades, J. M. (1984). Soil organic matter and structural stability: Mechanisms and implications for management. *Plant and Soil,**76*, 319–337.

[CR35] Polláková, N., Šimanský, V., & Kravka, M. (2018). The influence of soil organic matter fractions on aggregates stabilization in agricultural and forest soils of selected Slovak and Czech hilly lands. *Journal of Soils and Sediments,**18*, 2790–2800.

[CR36] Sharma, P. (2024). Biochar application for sustainable soil erosion control: A review of current research and future perspectives. *Frontiers in Environmental Science,**12*, 1373287.

[CR37] Shen, Z. (2024). An overview of biochar application in soil to immobilize heavy metals. In Z. Shen (Ed.), *An overview of biochar application in soil to immobilize heavy metals. Fundamental and casde studies* (pp. 1–7). Elsevier.

[CR38] Siebers, N., Voggenreiter, E., Joshi, P., Rethemeyer, J., & Wang, L. (2024). Synergistic relationships between the age of soil organic matter, Fe speciation, and aggregate stability in an arable Luvisol. *Journal of Plant Nutrition and Soil Science,**187*, 77–88.

[CR39] Šimanský, V. (2014). Short communication to the determination of soil structure. *Acta Fytotechnica Et Zootechnica,**17*, 1–5.

[CR40] Šimanský, V. (2016). Effects of biochar and biochar with nitrogen on soil organic matter and soil structure in Haplic Luvisol. *Acta Fytotechnica Et Zootechnica,**19*, 129–138.

[CR41] Šimanský, V., Šrank, D., Jonczak, J., & Juriga, M. (2019). Fertilization and application of different biochar types and their mutual interactions influencing changes of soil characteristics in soils of different textures. *Journal of Ecological Engineering,**20*, 149–164.

[CR42] Šimanský V, Polláková N, Chlpík J, Kolenčík M (2023a) Soil science. SPU, Nitra, Slovakia. (in Slovak).

[CR43] Šimanský, V., Wójcik-Gront, E., Rustowska, B., Juriga, M., Chlpík, J., & Macák, M. (2023b). Reducing machine movement intensity in the field improves soil structure. *Acta Fytotechnica Et Zootechnica,**26*, 93–101.

[CR44] Šrank, D., & Šimanský, V. (2020). Differences in soil organic matter and humus of sandy soil after application of biochar substrates and combination of biochar substrates with mineral fertilisers. *Acta Fytotechnica Et Zootechnica,**23*, 117–124.

[CR45] Stevenson, J. F. (1994). *Humus chemistry*. Wiley.

[CR46] Weber, J. (2020). Humic substances and their role in the environment. *EC Agriculture,**1*, 3–8.

[CR47] Weil, R. R., & Brady, N. C. (2017). *The nature and properties of soils*. Pearson Education Limited.

[CR48] Xu, G., Sun, J., Shao, H., & Chang, S. X. (2014). Biochar had effects on phosphorus sorption and desorption in three soils with differing acidity. *Ecological Engineering,**62*, 54–60.

[CR49] Zhang, J., Lü, F., Shao, L., & He, P. (2014). The use of biochar-amended composting to improve the humification and degradation of sewage sludge. *Bioresource Technology,**168*, 252–258. 24656550 10.1016/j.biortech.2014.02.080

[CR50] Zhang, J., Zhang, S., Niu, C., Jiang, J., & Sun, H. (2022). Positive effects of biochar on the degraded forest soil and tree growth in China: A systematic review. *Phyton,**91*(8), 1601–1616.

[CR51] Zhao, S., Ta, N., Li, Z., Yang, Y., Zhang, X., Liu, D., Zhang, A., & Wang, X. (2017). Varying pyrolysis temperature impacts application effects of biochar on soil labile organic carbon and humic substances. *Applied Soil Ecology,**116*, 399–409.

